# Antithrombotic drugs do not increase intraoperative blood loss in emergency gastrointestinal surgery: a single-institution propensity score analysis

**DOI:** 10.1186/s13017-019-0284-8

**Published:** 2019-12-30

**Authors:** Tadashi Matsuoka, Kenji Kobayashi, Alan Kawarai Lefor, Junichi Sasaki, Hiroharu Shinozaki

**Affiliations:** 1 Department of Surgery, Saiseikai Utsunomiya Hospital, Tochigi, Japan; 2 Department of Emergency and Critical Care Medicine, School of Medicine, Keio University, Tokyo, Japan; 3 Department of Surgery, Jichi Medical University, Tochigi, Japan

**Keywords:** Emergency gastrointestinal surgery, Antithrombotic drug, Intraoperative blood loss

## Abstract

**Background:**

The use of antithrombotic drugs is increasing with the aging population. Prior to elective procedures, antithrombotic drugs are often discontinued. For emergency procedures in patients taking antithrombotic drugs, their effect cannot be attenuated which may lead to an increased risk of hemorrhagic events. However, there are few studies showing increased intraoperative blood loss in patients taking antithrombotic drugs who undergo emergency gastrointestinal surgery. The aim of this study is to determine whether the use of antithrombotic agents increases intraoperative blood loss in emergency gastrointestinal surgery.

**Methods:**

A retrospective review of patients who underwent emergency abdominal surgery between January 2013 and December 2017 was conducted. The primary outcome measure was intraoperative blood loss. Patients were divided into the antithrombotic drug group and a control group, and a propensity score was developed using multivariate logistic regression. We use 1:1 propensity score matching analysis to compare outcomes between the two groups.

**Results:**

Of 1555 patients included in this study, 1184 patients, including 170 patients taking antithrombotic drugs, were eligible for propensity score matching analysis. A 1:1 matching yielded 117 well-balanced pairs. There was no statistically significant difference in intraoperative blood loss (antithrombotic drug group vs control group, median (interquartile): 60 (225–10) vs 100 (243–10) ml, *p* = 0.43).

**Conclusions:**

This study suggests that antithrombotic drugs do not increase intraoperative blood loss in patients undergoing emergency gastrointestinal surgery. Emergency gastrointestinal surgery for patients currently taking antithrombotic drugs can be performed safely, and the use of antithrombotic drugs is not a reason to delay surgical intervention.

## Background

Antithrombotic drugs have important prophylactic and therapeutic effects for patients with various diseases such as coronary artery disease [1], atrial fibrillation [2], cerebrovascular disease [3], and peripheral vascular disease [4]. These beneficial effects have been confirmed in many studies [5–8]. However, these medications can lead to bleeding which is an adverse effect [9, 10]. They increase the risk of cerebral hemorrhage [10] and gastrointestinal bleeding [11]. There are no agents to rapidly reverse the antithrombotic effects of many of these drugs. Therefore, they are usually stopped prior to the conduct of invasive procedures, such as surgery [12, 13] or endoscopic procedures [14–16].

In recent years, the number of patients taking anti-thrombotic drugs such as antiplatelet agents and anticoagulants is increasing along with the aging population [17]. Accordingly, surgeons must manage more patients taking these medications appropriately in the perioperative period. For elective surgery, stopping the antithrombotic drugs is recommended prior to the procedure in many situations [12, 13, 18]. Emergency surgery is often performed under the sustained effects of antithrombotic drugs. Some believe that use of antithrombotic drugs throughout the perioperative period might lead to increased intraoperative blood loss and postoperative bleeding [12–14, 19]. As one notable exception, it was reported that clopidogrel use might not lead to an increased incidence of postoperative bleeding events in abdominal surgery [20]. There is little evidence about the effect of antithrombotic drugs on intraoperative blood loss or the need for blood transfusion. Surgeons have great interest in these effects because they directly relate to intraoperative and postoperative management.

The purpose of this study was to evaluate whether antithrombotic drugs affect intraoperative blood loss in patients undergoing emergency gastrointestinal abdominal surgery. We hypothesized that antithrombotic drugs do not significantly increase intraoperative blood loss. This information is of great importance to surgeons who perform emergency abdominal surgery.

## Methods

### Study design

This retrospective study was approved by the Institutional Review Board of Saiseikai Utsunomiya Hospital (No.2018-16). Consecutive patients undergoing emergency abdominal surgery from January 2013 to December 2017 at Saiseikai Utsunomiya Hospital in Tochigi, Japan, were included in this study. Emergency gastrointestinal surgery was defined as operations performed within 24 h of arrival or performed due to deterioration after emergency admission and before planned elective surgery. Patients with traumatic injuries, those undergoing removal of a foreign body, or surgery for post-operative bleeding were excluded. Patients undergoing less frequently performed operations such as liver resection, pancreas resection, or splenectomy were also excluded. After excluding patients undergoing the operations listed above, all emergency gastrointestinal surgery in this study was classified into 1 of 7 types: gastrectomy, patch repair of a duodenal ulcer (e.g., Graham patch), intestinal surgery, colorectal surgery, stoma creation, appendectomy, or cholecystectomy. Clinical and demographic data for included patients were abstracted from the medical records.

### Antithrombotic drugs

Patients taking antiplatelet drugs and/or anticoagulants were classified in the antithrombotic drug group. Antiplatelet drugs included aspirin, clopidogrel, and others. Anticoagulants included warfarin, dabigatran, rivaroxaban, and apixaban. If patients stopped taking the antithrombotic drug before the start of a prescribed cessation period [16], they were considered off the drug. If patients stopped the antithrombotic drug within the prescribed cessation period, they were considered to be taking the drug and classified in the antithrombotic drug group. Patients not taking antithrombotic drugs were classified in the control group.

### Outcomes

The primary outcome of this study was intraoperative blood loss. Intraoperative blood loss was quantified by measuring suction fluid and weighing surgical gauzes used for blood and fluid collection, in which fluid other than blood such as ascites was subtracted. Secondary outcomes were postoperative bleeding and thrombotic events, the need for blood products, mortality, length of hospital stay, and postoperative complications. Bleeding events are defined as bleeding events due to any cause such as surgical site bleeding (superficial, deep, organ-space), operative site non-related gastrointestinal tract bleeding, and intracranial bleeding. Thrombotic events are defined as thrombotic events due to any cause such as myocardial infarction/unstable angina pectoris, intracranial infarction/transit ischemic attack, and venous thromboembolism (pulmonary embolism/deep vein thrombosis). The need for blood products is defined as the administration of blood products within the period from the time of surgery until 1 week postoperatively. Severe hemorrhage was defined as intraoperative massive bleeding (blood loss > 750 ml) or the administration of red blood cells because of intraoperative blood loss.

### Statistical analysis

#### Descriptive and bivariate analysis

All variables are expressed as the median (interquartile range (IQR)) or proportions. Baseline characteristics were compared between the antithrombotic drug group and the control group using the Mann-Whitney *U* test and Fisher’s exact test. Baseline characteristics and antithrombotic drug use were compared between patients with and without severe hemorrhage.

#### Multivariable logistic regression

Multivariate analyses were performed using logistic regression to identify independent risk factors for severe bleeding. Logistic regression was also used to determine any association of antithrombotic drug use with severe bleeding after controlling for potential confounders (the independent risk factor for severe bleeding).

#### Propensity score matching

Logistic regression analysis was used to estimate propensity scores to predict the use of antithrombotic drugs from available confounding factors. These factors included age, gender, and surgery type, which were chosen for their potential association with the outcome of interest based on clinical considerations. We did not select comorbidities as confounding factors because the antithrombotic drug group generally has more comorbidities such as coronary artery disease, arrhythmia, or cerebral infarction, which require treatment with antithrombotic drugs. If these comorbidities were set in the propensity score, groups after propensity score matching would be very small and markedly imbalanced. We performed propensity score matching using the following algorithm: 1:1 nearest neighbor matching with no replacement. We used a structured iterative approach to refine this logistic regression model to achieve a balance of covariates within the matched pairs. Standardized differences were used to measure covariate balance, whereby a standardized mean difference (SMD) above 10% represents a meaningful imbalance. After propensity score matching, the Mann-Whitney *U* and Fisher’s exact tests were used to explore differences in the two groups for continuous variables and categorical variables, respectively.

Differences were considered significant with a *p* value < 0.05. All data were analyzed using SPSS 26.0 statistical software (SPSS Inc., Chicago, IL).

## Results

### Patient characteristics

**Fig. 1:**
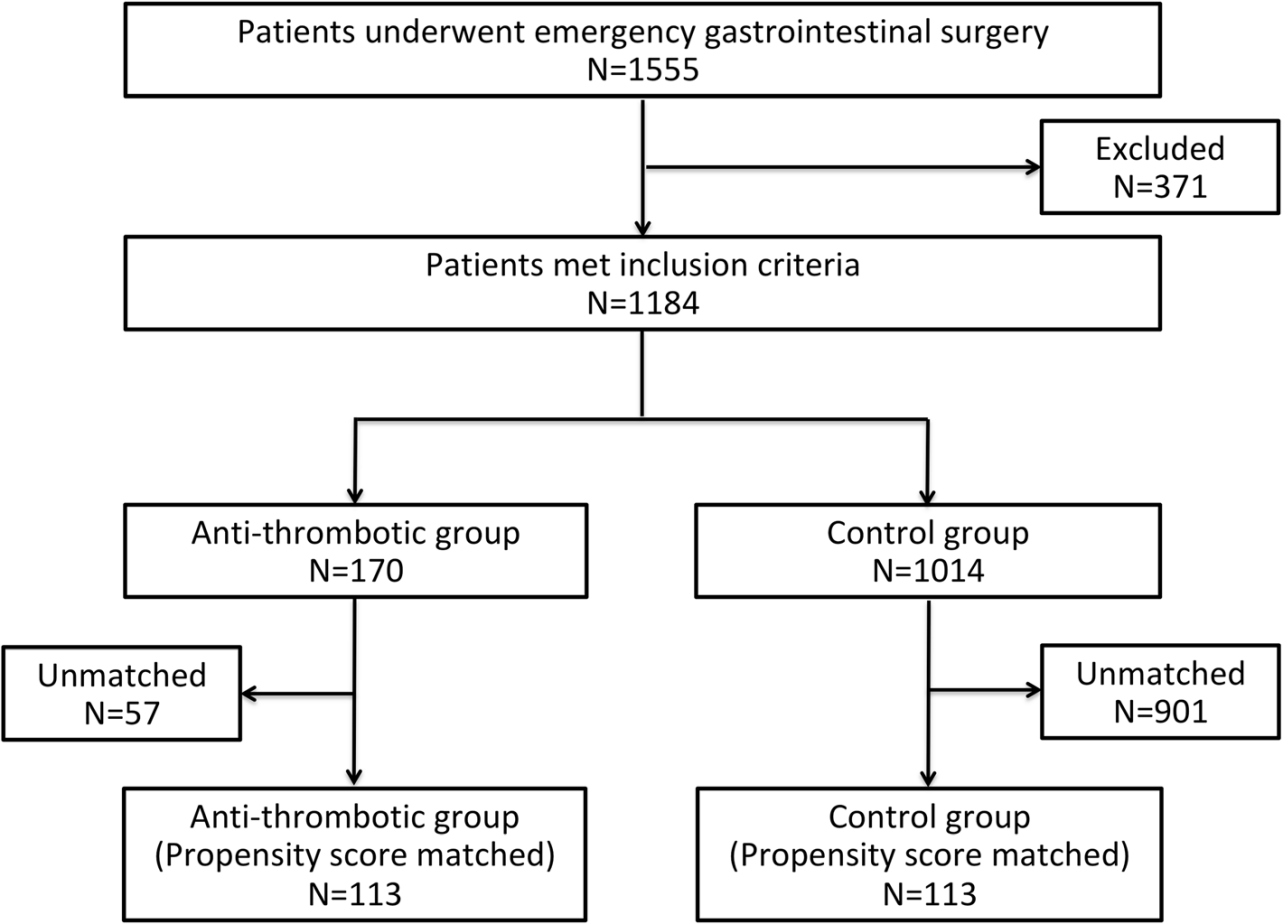
Study flow chart

During the study period, 1555 patients underwent emergency gastrointestinal surgery. After applying exclusion criteria (371 patients), 1184 patients remained and were analyzed as an unmatched cohort. Of these, 170 patients (14.4%) were taking antithrombotic drug at the time of emergency gastrointestinal surgery. Propensity score matching selected 113 patients who used antithrombotic drugs and 113 patients who did not (Fig. 1). Demographic and clinical characteristics before and after propensity score matching and antithrombotic drugs used are shown in Tables 1 and 2. Before matching, patients taking antithrombotic drugs were older and had more comorbidities such as diabetes mellitus, coronary artery disease, and hypertension. There were significant differences in the distribution of the types of surgery. Patients taking antithrombotic drugs were more likely to have undergone intestinal and colorectal surgery, and patients not taking antithrombotic drugs were more likely to have undergone an appendectomy. Laparotomy was more commonly performed for the emergency gastrointestinal procedure on patients taking antithrombotic drugs. After matching, variables such as age, gender, type of surgery, and surgical approach were well balanced between the two groups. The exception was the rate of comorbidities. Since comorbidities were not included in the estimation of the propensity score, there were differences of SMD > 0.1 after matching.

**Table 1:** Types of antithrombotic drugs and antidotes given

	Number	(%)
Antithrombotic drugs	170	100
Antiplatelet drugs	135	79
Aspirin	73	43
Clopidogrel	27	16
Other antiplatelet drugs	47	28
Anticoagulant drugs	42	25
Warfarin	24	14
Direct oral anticoagulants	18	11
Antidotes		
Vitamin K	7	4

**Table 2:** Demographic and clinical characteristics

	Before matching			After matching		
	AT	Control	SMD	AT	Control	SMD
Subjects	170	1014		113	113	
Age, years (range)	79 (84–68)	59 (73–33)	1.17	77 (84–69)	77 (84–69)	0.00
Gender, male	101 (59.4)	573 (56.5)	0.04	73 (64.6)	73 (64.6)	0.00
Type of surgery						
Gastrectomy	1 (0.6)	11 (1.1)	− 0.14	1 (0.9)	1 (0.9)	0.00
Patch repair duodenal ulcer	5 (2.9)	56 (5.5)	− 0.05	3 (2.7)	3 (2.7)	0.00
Intestinal surgery	78 (45.9)	252 (24.9)	− 0.13	60 (53.1)	60 (53.1)	0.00
Colorectal surgery	42 (24.7)	129 (12.7)	0.45	28 (24.8)	28 (24.8)	0.00
Stoma creation	18 (10.6)	85 (8.4)	0.31	7 (6.2)	7 (6.2)	0.00
Appendectomy	23 (13.5)	437 (43.1)	0.08	13 (11.5)	13 (11.5)	0.00
Cholecystectomy	3 (1.8)	44 (4.3)	− 0.70	1 (0.9)	1 (0.9)	0.00
Surgical approach						
Laparotomy	154 (90.6)	60 (59.5)	− 0.15	103 (91.2)	104 (92.0)	− 0.03
Laparoscopy	16 (9.4)	411 (40.5)	0.77	10 (8.8)	9 (8.0)	0.03
Comorbidities						
Diabetes mellitus	43 (25.3)	87 (8.6)	0.46	27 (23.9)	21 (18.6)	0.13
Renal failure	21 (12.4)	30 (3.0)	0.36	12 (10.6)	4 (3.5)	0.3
Liver cirrhosis	6 (3.5)	20 (2.0)	0.09	3 (2.7)	3 (2.7)	0.00
Coronary artery disease	58 (34.1)	9 (0.9)	0.98	36 (31.9)	2 (1.8)	0.88
Atrial fibrillation	39 (22.9)	18 (1.8)	0.68	26 (23.0)	5 (4.4)	0.57
Cerebrovascular disease	46 (27.1)	11 (1.1)	0.81	33 (29.2)	4 (3.5)	0.74
Deep vein thrombosis	14 (8.2)	11 (1.1)	0.34	9 (8.0)	3 (2.7)	0.24
Hypertension	120 (70.6)	259 (25.5)	1.01	79 (69.9)	51 (45.1)	0.52
Malignancy	63 (37.1)	208 (20.5)	0.37	35 (31.0)	31 (27.4)	0.08

Data are presented as number (percentage) or median (interquartile)

*AT* antithrombotic drug group, *SMD* standardized mean difference

### Primary outcome

**Fig. 2:**
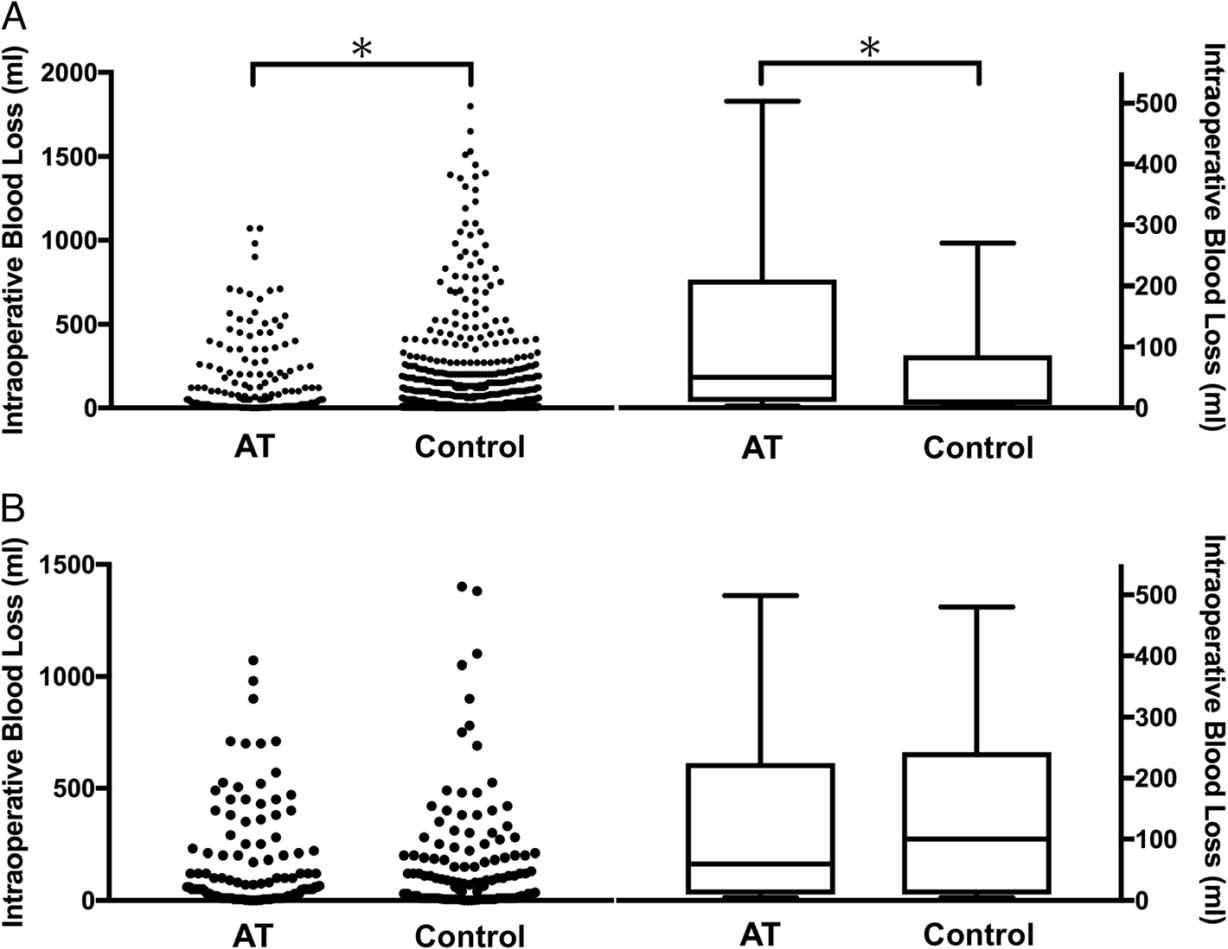
Comparison of intraoperative blood loss in analysis for antithrombotic drug use (dot plot/box plot). **a** Before matching. **b** After matching. *p < 0.05 compared with the control group as analyzed using Mann-Whitney *U* test. AT=antithrombotic drug group, Ctrl=control group

Before matching, intraoperative blood loss in the antithrombotic drug group was significantly greater than that in the control group (antithrombotic drug group vs control group, median (IQR) 50 (210–10)) vs 10 (86–5) ml, *p* < 0.001). However, after matching, intraoperative blood loss in the antithrombotic drug group was similar to that of the control group (60 (225–10) vs 100 (243–10) ml, *p* = 0.433) (Fig. 2, Table 3).

**Table 3:** Primary and secondary outcomes

	Before matching			After matching		
	AT	Control	*p* value	AT	Control	*p* value
Subjects	170	1014		113	113	
Intraoperative blood loss (ml)	50 (210–10)	10 (86–5)	< 0.001	60 (225–10)	100 (243–10)	0.433
Severe hemorrhage	33 (19.4)	137 (13.5)	0.03	25 (22.1)	22 (19.5)	0.743
Blood transfusion						
Red Blood Cell	36 (21.2)	123 (12.1)	0.002	27 (23.9)	18 (15.9)	0.182
Amount (units)	0 (0–0)	0 (0–0)	0.002	0 (0–0)	0 (0–0)	0.127
Fresh Frozen Plasma	30 (17.6)	74 (7.3)	< 0.001	19 (16.8)	15 (13.3)	0.577
Amount (units)	0 (0–0)	0 (0–0)	< 0.001	0 (0–0)	0 (0–0)	0.430
Platelet	8 (4.7)	22 (2.2)	0.063	7 (6.2)	2 (1.8)	0.171
Amount (units)	0 (0–0)	0 (0–0)	0.051	0 (0–0)	0 (0–0)	0.090
Bleeding events	7 (4.1)	25 (2.5)	0.206	5 (4.4)	2 (1.8)	0.446
Thrombotic events	4 (2.4)	21 (2.1)	0.773	2 (1.8)	3 (2.7)	1.000
Mortality	16 (9.4)	35 (3.5)	0.001	11 (9.7)	5 (4.4)	0.193
Operative time (min)	101 (149–73)	90 (127–65)	< 0.001	99 (150–72)	117 (151–77)	0.244
Length of stay (days)	21 (33–13)	6 (19–10)	< 0.001	21 (33–13)	16 (29–11)	0.060
Other complications						
Surgical site infection	33 (19.4)	133 (13.1)	0.032	26 (23.0)	29 (25.7)	0.757
Abscess	20 (11.8)	112 (11.0)	0.792	14 (12.4)	18 (15.9)	0.568
Pneumonia	21 (12.4)	43 (4.2)	< 0.001	15 (13.3)	9 (8.0)	0.280

Data are presented as number (percentage) or median (interquartile)

One unit of red blood cells, fresh frozen plasma, and platelet are approximately 120 cc, 120 cc, and 20 cc, respectively

*AT* antithrombotic drug group

### Secondary outcomes

Table 3 shows the results of secondary outcomes in this study. Before matching, variables such as the rate and volume of blood transfusions, presence of severe bleeding, mortality, operative time, length of stay, and rate of surgical site infection were higher in the antithrombotic drug group than in the control group. After matching, these variables for the antithrombotic group were similar to those of the control group. Table 4 shows the risk factors for severe bleeding, assessed by bivariate analyses. There were significant differences in age (severe bleeding vs non-severe bleeding 77 (84–64) vs 59 (73–33), *p* < 0.001), male (49.4% vs 58.0%, *p* = 0.037), antithrombotic drug use (19.4% vs 12.8%, *p* = 0.030), type of surgery, surgical approach, and comorbidities. As a result of analysis of these variables in the multivariate model, age (per 10 years) (odds ratio (OR) 1.28, 95% confidence interval (CI) (1.12–1.46)), gastrectomy (OR 6.77, 95% CI (1.73–26.50)), intestinal surgery (OR 0.43, 95% CI (0.26–0.71)), colorectal resection (OR 1.90, 95% CI (1.17–3.10)), appendectomy (OR 0.19, 95% CI (0.08–0.46)), laparotomy (OR 5.243, 95% CI (2.23–13.21)), renal failure (OR 2.59, 95% CI (1.35–4.99)), and malignancy (OR 1.55, 95% CI (1.05–2.29)) were independent risk factors (Table 5). Antithrombotic drug use was not an independent risk factor (antithrombotic drug (OR 0.73, 95% CI (0.45–1.17), *p* = 0.193), antiplatelet drugs (OR 0.71, 95% CI (0.42–1.21), *p* = 0.204), anticoagulant drugs (OR 1.29, 95% CI (0.61–2.71), *p* = 0.508), and dual antithrombotic drugs (OR 0.66, 95% CI (0.21–2.10), *p* = 0.480)).

**Table 4:** Bivariate analyses of severe bleeding

	Severe bleeding	Non severe bleeding	*p* value
Subjects	170	1014	
Age, years (range)	77 (84–64)	59 (73–33)	<0.001
Gender, male	84 (49.4)	588 (58.0)	0.037
Antithrombotic drugs	33 (19.4)	130 (12.8)	0.030
Antiplatelet drugs	23 (13.5)	105 (10.4)	0.230
Anticoagulant drugs	14 (8.2)	28 (2.8)	0.001
Dual antithrombotic drugs	4 (2.4)	22 (2.2)	0.781
Type of surgery			
Gastrectomy	9 (5.3)	3 (0.3)	< 0.001
Patch repair duodenal ulcer	7 (4.1)	54 (5.3)	0.707
Intestinal surgery	43 (25.3)	285 (28.1)	0.461
Colorectal surgery	67 (39.4)	104 (10.3)	< 0.001
Stoma formation	30 (17.6)	73 (7.2)	< 0.001
Appendectomy	7 (4.1)	453 (44.7)	<0.001
Cholecystectomy	7 (4.1)	40 (3.9)	0.834
Surgical approach			<0.001
Laparotomy	164 (96.5)	591 (58.3)	
Laparoscopy	6 (3.5)	423 (41.7)	
Comorbidities			
Diabetes mellitus	36 (21.2)	94 (9.3.6)	<0.001
Renal failure	20 (11.8)	30 (3.0)	<0.001
Liver cirrhosis	11 (6.5)	15 (1.5)	<0.001
Coronary artery disease	10 (5.9)	55 (5.4)	0.855
Atrial fibrillation	20 (11.8)	36 (3.6)	<0.001
Cerebrovascular disease	14 (8.2)	43 (4.2)	0.032
Deep vein thrombosis	7 (4.1)	18 (1.8)	0.075
Hypertension	97 (57.1)	280 (27.6)	<0.001
Malignancy	79 (46.5)	192 (18.9)	<0.001
Blood transfusion			
Red Blood Cell	159 (93.5)	0 (0.0)	<0.001
Amount (units)	4 (2–6)	0 (0–0)	<0.001
Fresh frozen plasma	81 (47.6)	24 (2.4)	<0.001
Amount (units)	0 (10–0)	0 (0–0)	<0.001
Platelet	27 (15.9)	4 (0.4)	<0.001
Amount (units)	0 (0–0)	0 (0–0)	<0.001
Bleeding events	21 (12.4)	12 (1.2)	<0.001
Thrombotic events	9 (5.3)	16 (1.6)	0.006
Mortality	36 (21.2)	14 (1.4)	<0.001
Operative time (min)	136 (183–100)	86 (121–63)	<0.001
Length of stay (days)	31 (53–17)	10 (18–5)	<0.001
Other complications			
Surgical site infection	57 (33.5)	108 (10.7)	<0.001
Abscess	50 (29.4)	81 (8.0)	<0.001
Pneumonia	35 (20.6)	28 (2.8)	<0.001

Data are presented as number (percentage) or median (interquartile). One unit of red blood cells, fresh frozen plasma, and platelet are approximately 120 cc, 120 cc, and 20 cc, respectively

**Table 5:** Multivariate analysis of severe bleeding

	Coefficient	*p* value	Odds ratio	95% confidential interval	
Age (per 10 years)	0.24	<0.001	1.28	1.12	1.46
Gastrectomy	1.91	0.006	6.77	1.73	26.50
Intestinal surgery	− 0.85	0.001	0.43	0.26	0.71
Colorectal surgery	0.64	0.010	1.90	1.17	3.10
Appendectomy	− 1.65	<0.001	0.19	0.08	0.46
Laparotomy	1.69	<0.001	5.43	2.23	13.21
Renal failure	0.95	0.004	2.59	1.35	4.99
Malignancy	0.44	0.029	1.55	1.05	2.29
Antithrombotic drug	− 0.32	0.193	0.73	0.45	1.17
Antiplatelet drug	− 0.34	0.204	0.71	0.42	1.21
Anticoagulant drug	0.25	0.508	1.29	0.61	2.71
Dual antithrombotic drug	− 0.42	0.480	0.66	0.21	2.10

*χ*^2^<0.001, AUC = 0.840 (0.813–0.868), percentage correct 86.6%

In the analysis of antiplatelet drugs and dual antithrombotic drugs, as well as the analysis of antithrombotic drug use, although patients in the antiplatelet drug group and dual antithrombotic drug group had more intraoperative blood loss than the control group before matching, the median blood loss in these groups was not statistically significantly different from the control group after matching (Additional file 1 (A, B), Additional file 2 (A, B), Additional file 3, and Additional file 4)

## Discussion

This study suggests that antithrombotic drugs have no significant effect on the volume of intraoperative blood loss in emergency gastrointestinal surgery after adjustment for confounding factors by propensity score matching. To the best of our knowledge, this is the first report to evaluate the relationship between antithrombotic drug use and intraoperative blood loss in patients undergoing emergency gastrointestinal surgery.

Increase of intraoperative blood loss confers unfavorable effects on immune function [21–23] and is associated with major complications or a worse prognosis in patients undergoing a variety of operations [24, 25]. Other studies reported that more intraoperative blood loss induces suppression of anti-tumor effects, microscopic spillage of cancer cells in the blood resulting in a worse prognosis in patients undergoing surgery for cancer [21, 26, 27]. These studies support the idea that a decrease in unnecessary bleeding results in less harm to patients undergoing gastrointestinal surgery. Patients currently taking antithrombotic drugs may be thought to have increased bleeding tendencies. Based on the results of this study, surgeons do not need to hesitate to perform surgery in these patients.

Before matching, patients using antithrombotic drugs had more intraoperative blood loss, higher rate of blood transfusions, higher mortality, longer hospital stay, and a higher rate of surgical site infections than patients not taking antithrombotic drugs. However, after adjustment for confounding factors, the outcomes were comparable between the two groups, with no significant differences. Patients taking antiplatelet drugs alone and those taking dual antithrombotic drugs had results similar to results for patients taking antithrombotic drugs. In multivariate analysis, the use of antithrombotic drugs, including antiplatelet drugs and dual antithrombotic drugs, was not an independent risk factor for severe bleeding. These results suggest that age and the type of surgery are related to intraoperative blood loss and other outcomes, but antithrombotic drug use is not related. The use of antithrombotic drugs alone does not seem to increase the risk of intraoperative blood loss, postoperative bleeding, or thrombotic events.

In the guidelines for gastroenterological endoscopy in patients currently receiving antithrombotic treatment [15, 16], withdrawal of aspirin monotherapy is not required for patients who would be placed at high risk of thromboembolism by cessation. It is recommended that in patients with a low risk of thromboembolism, aspirin can be withdrawn for 3 to 5 days [15, 16]. In elective general and abdominal surgery for patients not at high risk of cardiovascular events, Antolovic et al. reported that continuation of an antiplatelet drug did not influence the incidence of severe bleeding [28]. There was no difference in intraoperative blood loss, postoperative anemia, or blood transfusion requirement for patients with and without aspirin therapy undergoing laparoscopic cholecystectomy [29]. While some studies reviewed very specific patient, disease, or surgery types, there are few evidence-based studies of the relationship between antithrombotic drugs and emergency gastrointestinal surgery reviewing a wide range of patients and diseases. Recently, Jupiter et al. reported the relationship between clopidogrel use and postoperative bleeding [20]. These investigators concluded that clopidogrel use slightly increases postoperative bleeding events statistically, but has no significant clinical effect. At the time of that study, whether antithrombotic drugs actually increase intraoperative blood loss or postoperative thrombotic events had not been shown. This led to difficulties in assessing the risk of increased intraoperative blood loss or the need for blood transfusions in emergency gastrointestinal surgery for patients taking antithrombotic drugs. The present study suggests that antithrombotic drugs do not increase intraoperative blood loss, need for blood transfusion, or postoperative bleeding and thrombotic events. Since the perioperative risk for patients undergoing emergency surgery is higher than patients undergoing elective surgery, these results may apply to elective procedures as well but further study is needed.

This study has acknowledged limitations. First, although propensity score matching is used to decrease the bias between the two groups, this study is retrospective. Propensity score matching cannot equalize unmeasured confounding factors, so there might be residual confounders affecting these results. There are some differences in comorbidities between the two groups after propensity matching. Therefore, unmeasured confounding factors and differences in comorbidities may affect these results. Second, the study is not blinded. With the information regarding antithrombotic drug use, surgeons may be especially careful with the management of blood loss. Third, the use of antidotes and the timing of restarting antithrombotic drugs were left to the discretion of each attending surgeon. Vitamin K, which needs time to normalize the PT-INR, was given only to a few patients, and if vitamin K was given, it would likely not be effective as an antidote during the operation. Therefore, we think the effect of vitamin K on the results of this study is minimal. Surgeons tend to restart antithrombotic drugs when they believe the risk of postoperative bleeding has subsided. Without a uniform standard for postoperative resumption of antithrombotic drugs, there is variation. Fourth, because the sample size became small due to propensity score matching, we might not have been able to find a significant difference in this study. Fifth, because the effect of combinations of antithrombotic drugs and the type of surgery were not analyzed, the heterogeneity of antithrombotic drugs given and type of surgery might affect the results. Finally, the judgment to perform the operation and the choice of procedure depends on each surgeon. For a patient taking antithrombotic drugs with a high risk of bleeding, surgeons might choose a less invasive procedure, or non-operative therapy, which they would not choose if the patient did not use antithrombotic drugs.

## Conclusion

This study suggests that antithrombotic drugs do not significantly affect intraoperative blood loss in patients undergoing emergency gastrointestinal surgery. These findings should improve the approach to patients taking antithrombotic drug, who need emergency gastrointestinal surgery. A large-scale retrospective study or a prospective randomized controlled trial is required to confirm these findings.

## Supplementary information

**Additional file 1.** Comparison of intraoperative blood loss in analysis for antiplatelet drug use (dot plot / box plot). (A) before matching, (B) after matching. Description: *P<0.05 compared with the control group as analyzed Mann Whitney U test. AP=antiplatelet drug group, Ctrl=control group.

**Additional file 2.** Comparison of intraoperative blood loss in analysis for dual antithrombotic drug use (dot plot / box plot). (A) before matching, (B) after matching. Description: DAT=antithrombotic drug group

**Additional file 3.** Demographic and Clinical Characteristics for antiplatelet drug analysis. Description: Data are presented as number (percentage) or median (interquartile). AP=antiplatelet drug group; SMD=standardized mean difference.

**Additional file 4.** Demographic and Clinical Characteristics for dual antithrombotic drug analysis. Description: Data are presented as number (percentage) or median (interquartile). DAT=dual antithrombotic drug group; SMD=standardized mean difference.

## Data Availability

The datasets used and/or analyzed during the current study are available from the corresponding author on reasonable request.

## Abbreviations

CI: Confidence interval
IQR: Interquartile range
OR: Odds ratio
SMD: Standardized mean difference
