# Supplementary figures and images for: Antithrombotic drugs do not increase intraoperative blood loss in emergency gastrointestinal surgery: a single-institution propensity score analysis
Source: World J Emerg Surg. 2019 Dec 30;14:63. doi: 10.1186/s13017-019-0284-8 (PMC6938014; doi:10.1186/s13017-019-0284-8)

A

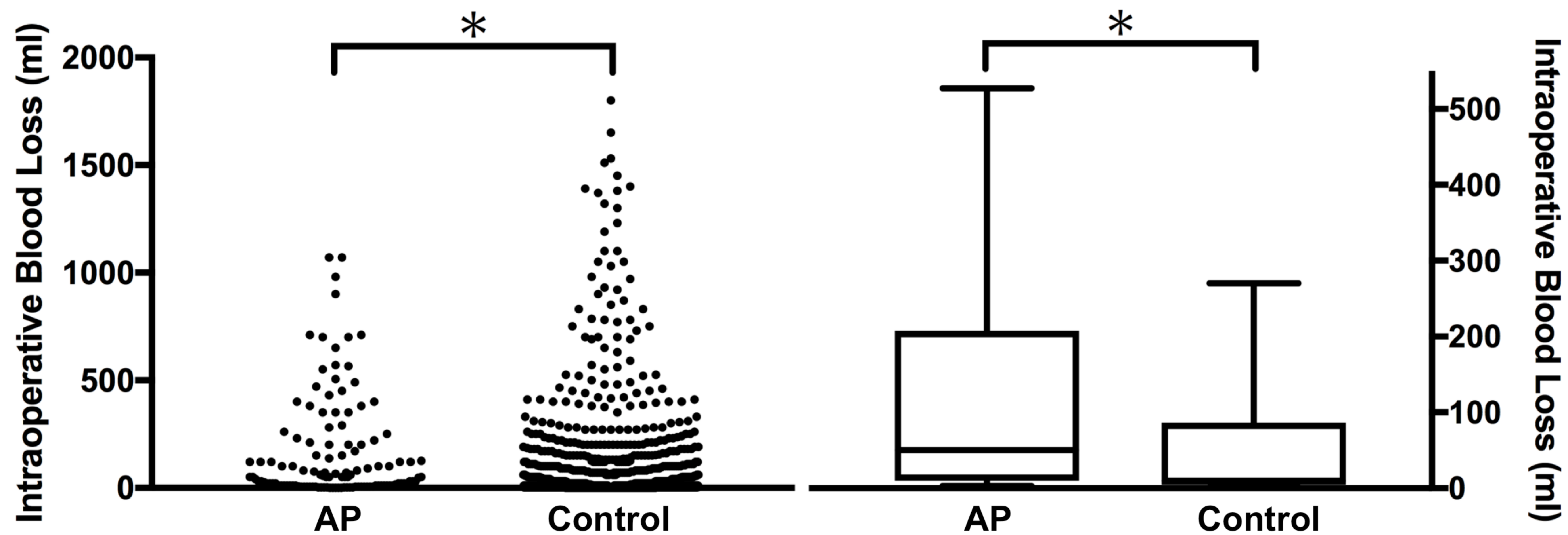

B

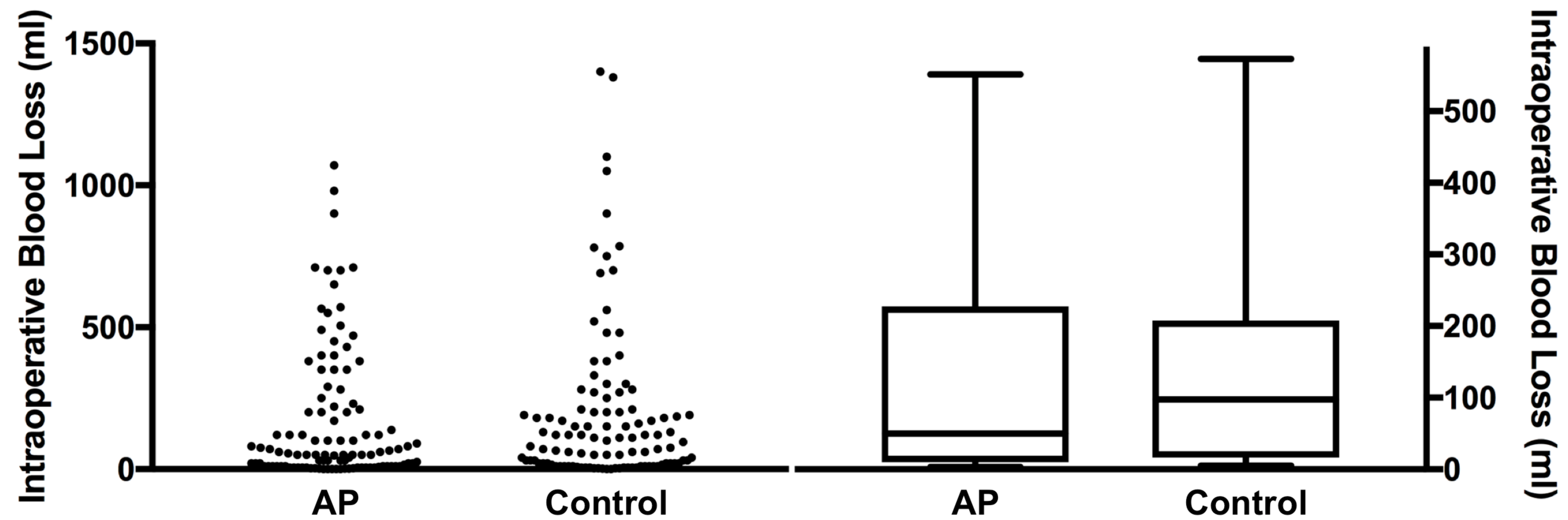

Supplement: Supplementary file 1 — Additional file 1. Comparison of intraoperative blood loss in analysis for antiplatelet drug use (dot plot / box plot). (A) before matching, (B) after matching. Description: *P<0.05 compared with the control group as analyzed Mann Whitney U test. AP=antiplatelet drug group, Ctrl=control group. [file 13017_2019_284_MOESM1_ESM.pdf]

A

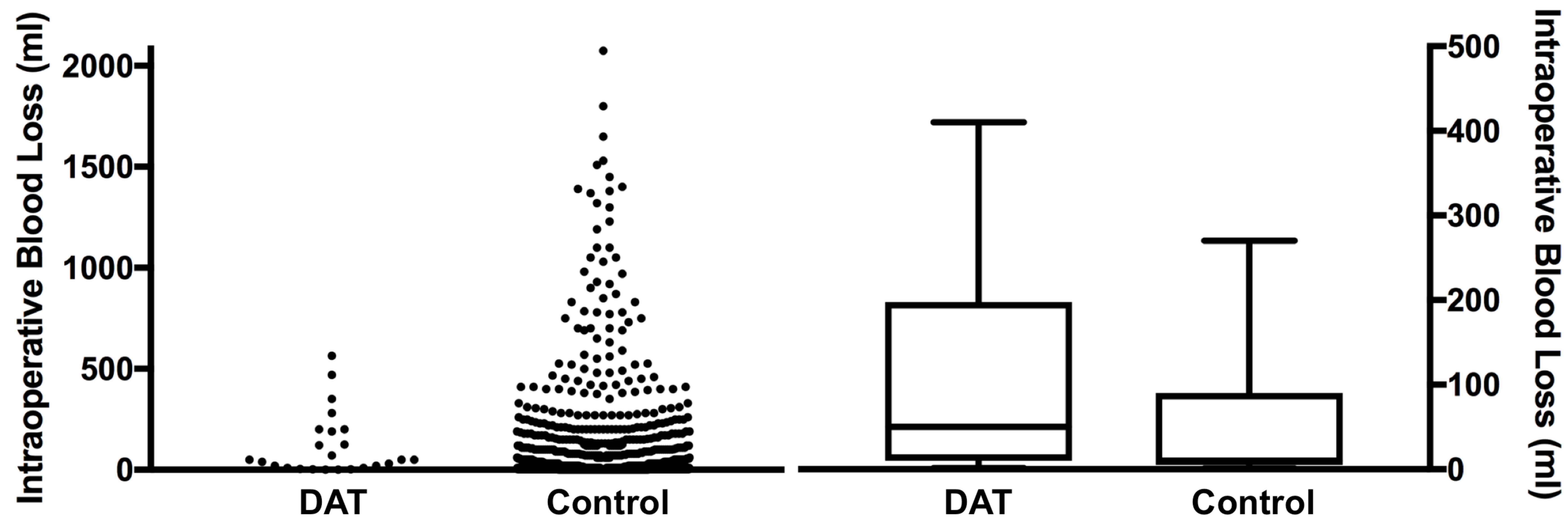

B

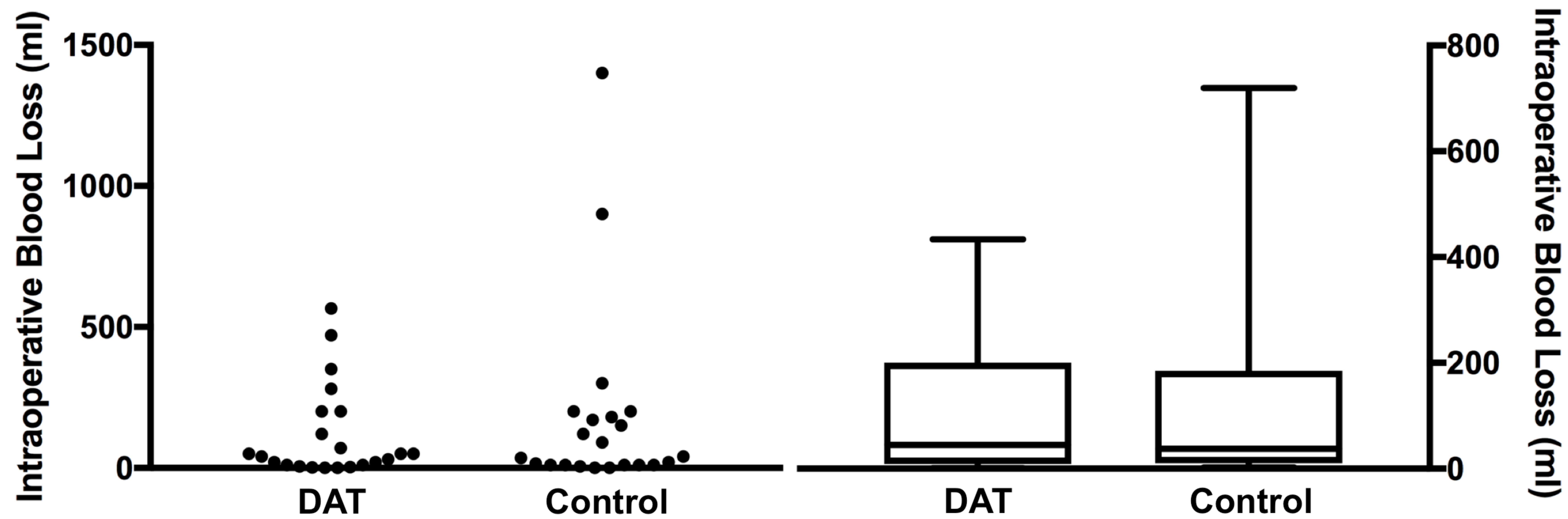

Supplement: Supplementary file 2 — Additional file 2. Comparison of intraoperative blood loss in analysis for dual antithrombotic drug use (dot plot / box plot). (A) before matching, (B) after matching. Description: DAT=antithrombotic drug group [file 13017_2019_284_MOESM2_ESM.pdf]
