# Supplementary material for: Antithrombotic drugs do not increase intraoperative blood loss in emergency gastrointestinal surgery: a single-institution propensity score analysis
Source: World J Emerg Surg. 2019 Dec 30;14:63. doi: 10.1186/s13017-019-0284-8 (PMC6938014; doi:10.1186/s13017-019-0284-8)
Supplement: Supplementary file 3 — Additional file 3. Demographic and Clinical Characteristics for antiplatelet drug analysis. Description: Data are presented as number (percentage) or median (interquartile). AP=antiplatelet drug group; SMD=standardized mean difference. [file 13017_2019_284_MOESM3_ESM.docx]

**Additional file 3.** Demographic and Clinical Characteristics for antiplatelet drug analysis.

|  | Before Matching | | |  | After Matching | | |
| --- | --- | --- | --- | --- | --- | --- | --- |
|  | AP | Control | SMD |  | AP | Control | SMD |
| Subjects | 124 | 1014 |  |  | 108 | 108 |  |
| Age, years (range) | 76 (85-67) | 59 (73-33) | 1.17 |  | 77 (84-67) | 77 (84-67) | 0.00 |
| Gender, Male | 77 (62.1) | 573 (56.5) | 0.11 |  | 70 (64.8) | 75 (69.4) | -0.10 |
| Type of surgery |  |  |  |  |  |  |  |
| Gastrectomy | 0 (0.0) | 11 (1.1) | -0.15 |  | 0 (0.0) | 0 (0.0) | 0.00 |
| Patch repair duodenal ulcer | 5 (4.0) | 56 (5.5) | -0.30 |  | 4 (3.7) | 4 (3.7) | 0.00 |
| Intestinal surgery | 59 (47.6) | 252 (24.9) | 0.49 |  | 55 (50.9) | 55 (50.9) | 0.00 |
| Colorectal surgery | 28 (22.6) | 129 (12.7) | 0.26 |  | 26 (24.1) | 26 (24.1) | 0.00 |
| Stoma formation | 11 (8.9) | 85 (8.4) | 0.02 |  | 8 (7.4) | 8 (7.4) | 0.00 |
| Appendectomy | 19 (15.3) | 437 (43.1) | -0.64 |  | 14 (13.0) | 14 (13.0) | 0.00 |
| Cholecystectomy | 2 (1.6) | 44 (4.3) | -0.16 |  | 1 (0.9) | 1 (0.9) | 0.00 |
| Surgical approach |  |  |  |  |  |  |  |
| Laparotomy | 110 (88.7) | 60 (59.5) | 0.71 |  | 96 (88.9) | 98 (90.7) | -0.06 |
| Laparoscopy | 14 (11.3) | 411 (40.5) | -0.93 |  | 12 (11.1) | 10 (9.3) | 0.06 |
| Comorbidities |  |  |  |  |  |  |  |
| Diabetes mellitus | 31 (25.0) | 87 (8.6) | 0.45 |  | 27 (25.0) | 20 (18.5) | 0.16 |
| Renal failure | 12 (9.7) | 30 (3.0) | 0.28 |  | 12 (11.1) | 5 (4.6) | 0.24 |
| Liver cirrhosis | 4 (3.2) | 20 (2.0) | 0.08 |  | 3 (2.8) | 2 (1.9) | 0.06 |
| Coronary artery disease | 49 (39.5) | 9 (0.9) | 1.10 |  | 43 (39.8) | 3 (2.8) | 1.01 |
| Atrial fibrillation | 4 (3.2) | 18 (1.8) | 0.09 |  | 4 (3.7) | 4 (3.7) | 0.00 |
| Cerebrovascular disease | 37 (29.8) | 11 (1.1) | 0.87 |  | 33 (30.6) | 4 (3.7) | 0.76 |
| Deep vein thrombosis | 7 (5.6) | 11 (1.1) | 0.25 |  | 6 (5.6) | 6 (5.6) | 0.00 |
| Hypertension | 93 (75.0) | 259 (25.5) | 1.14 |  | 79 (73.1) | 44 (40.7) | 0.70 |
| Malignancy | 42 (33.9) | 208 (20.5) | 0.30 |  | 35 (32.4) | 32 (29.6) | 0.06 |

Data are presented as number (percentage) or median (interquartile).

AP, antiplatelet drug group; SMD, standardized mean difference.
