# Supplementary material for: Antithrombotic drugs do not increase intraoperative blood loss in emergency gastrointestinal surgery: a single-institution propensity score analysis
Source: World J Emerg Surg. 2019 Dec 30;14:63. doi: 10.1186/s13017-019-0284-8 (PMC6938014; doi:10.1186/s13017-019-0284-8)
Supplement: Supplementary file 4 — Additional file 4. Demographic and Clinical Characteristics for dual antithrombotic drug analysis. Description: Data are presented as number (percentage) or median (interquartile). DAT=dual antithrombotic drug group; SMD=standardized mean difference. [file 13017_2019_284_MOESM4_ESM.docx]

**Additional file 4.** Demographic and Clinical Characteristics for dual antithrombotic drug analysis

|  | Before Matching | | |  | After Matching | | |
| --- | --- | --- | --- | --- | --- | --- | --- |
|  | DAT | Control | SMD |  | DAT | Control | SMD |
| Subjects | 24 | 1014 |  |  | 22 | 22 |  |
| Age, years (range) | 77 (81-67) | 59 (73-33) | 1.17 |  | 75 (81-67) | 75 (81-67) | 0.00 |
| Gender, Male | 16 (66.7) | 573 (56.5) | 0.11 |  | 15 (68.2) | 15 (68.2) | 0.00 |
| Type of surgery |  |  |  |  |  |  |  |
| Gastrectomy | 0 (0.0) | 11 (1.1) | -0.15 |  | 0 (0.0) | 0 (0.0) | 0.00 |
| Patch repair duodenal ulcer | 0 (0.0) | 56 (5.5) | -0.30 |  | 0 (0.0) | 0 (0.0) | 0.00 |
| Intestinal surgery | 17 (70.8) | 252 (24.9) | 0.49 |  | 15 (68.2) | 15 (68.2) | 0.00 |
| Colorectal surgery | 3 (12.5) | 129 (12.7) | 0.26 |  | 3 (13.6) | 3 (13.6) | 0.00 |
| Stoma formation | 3 (12.5) | 85 (8.4) | 0.02 |  | 3 (13.6) | 3 (13.6) | 0.00 |
| Appendectomy | 1 (4.2) | 437 (43.1) | -0.64 |  | 1 (4.5) | 1 (4.5) | 0.00 |
| Cholecystectomy | 0 (0.0) | 44 (4.3) | -0.16 |  | 0 (0.0) | 0 (0.0) | 0.00 |
| Surgical approach |  |  |  |  |  |  |  |
| Laparotomy | 23 (95.8) | 60 (59.5) | 0.71 |  | 21 (95.5) | 21 (95.5) | 0.00 |
| Laparoscopy | 1 (4.2) | 411 (40.5) | -0.93 |  | 1 (0.5) | 1 (0.5) | 0.00 |
| Comorbidities |  |  |  |  |  |  |  |
| Diabetes mellitus | 7 (29.2) | 87 (8.6) | 0.45 |  | 6 (27.3) | 6 (27.3) | 0.00 |
| Renal failure | 3 (12.5) | 30 (3.0) | 0.28 |  | 2 (9.1) | 1 (4.5) | 0.18 |
| Liver cirrhosis | 2 (8.3) | 20 (2.0) | 0.08 |  | 1 (4.5) | 1 (4.5) | 0.00 |
| Coronary artery disease | 11 (45.8) | 9 (0.9) | 1.10 |  | 10 (45.5) | 0 (0.0) | 1.3 |
| Atrial fibrillation | 7 (29.2) | 18 (1.8) | 0.09 |  | 6 (27.3) | 0 (0.0) | 0.87 |
| Cerebrovascular disease | 7 (29.2) | 11 (1.1) | 0.87 |  | 6 (27.3) | 0 (0.0) | 0.87 |
| Deep vein thrombosis | 2 (8.3) | 11 (1.1) | 0.25 |  | 2 (9.1) | 1 (4.5) | 0.18 |
| Hypertension | 17 (70.8) | 259 (25.5) | 1.14 |  | 15 (68.2) | 10 (45.5) | 0.47 |
| Malignancy | 7 (29.2) | 208 (20.5) | 0.30 |  | 6 (27.3) | 10 (45.5) | -0.39 |

Data are presented as number (percentage) or median (interquartile).

DAT, dual antithrombotic drug group; SMD, standardized mean difference.
